# Conflicting evidence for the role of JNK as a target in breast cancer cell proliferation: Comparisons between pharmacological inhibition and selective shRNA knockdown approaches

**DOI:** 10.1002/prp2.376

**Published:** 2017-12-20

**Authors:** Rachel A. Wood, Mark J. Barbour, Gwyn W. Gould, Margaret R. Cunningham, Robin J. Plevin

**Affiliations:** ^1^ Strathclyde Institute for Pharmacy and Biomedical Sciences (SIPBS) University of Strathclyde Glasgow UK; ^2^ Institute of Molecular, Cell and Systems Biology College of Medical, Veterinary and Life Sciences University of Glasgow Glasgow UK

**Keywords:** breast cancer, JNK, Lentivirus, MAPK, MCF‐7, SP600125

## Abstract

As a target, the JNK pathway has been implicated in roles including cell death, proliferation, and inflammation in variety of contexts which span cardiovascular disease, neurodegenerative pathologies, and cancer. JNK1 and JNK2 have recently been demonstrated to function independently, highlighting a new parameter in the study of the JNK pathway. In order for JNK1 and JNK2‐specific roles to be defined, better tools need to be employed. Previous studies have relied upon the broad spectrum JNK inhibitor, SP600125, to characterize the role of JNK signaling in a number of cell lines, including the breast cancer cell line MCF‐7. In line with previous literature, our study has demonstrated that SP600125 treatment inhibited c‐Jun and JNK phosphorylation and MCF‐7 proliferation. However, in addition to targeting JNK1, JNK2, and JNK3, SP600125 has been previously demonstrated to suppress the activity of a number of other serine/threonine kinases, making SP600125 an inadequate tool for JNK isoform‐specific roles to be determined. In this study, lentiviral shRNA was employed to selectively knockdown JNK1, JNK2, and JNK1/2 in MCF‐7 cells. Using this approach, JNK phosphorylation was fully inhibited following stable knockdown of respective JNK isoforms. Interestingly, despite suppression of JNK phosphorylation, MCF‐7 cell proliferation, cell cycle progression, or cell death remained unaffected. These findings raise the question of whether JNK phosphorylation really is pivotal in MCF‐7 cell growth and death or if suppression of these events is a result of one of the many off‐targets cited for SP600125.

**Abbreviations**
ANOVA: analysis of variance
BSA: Bovine Serum Albium
HCC: hepatocellular carcinoma
JNK: Jun N‐terminal kinase
MEFs: mouse embryonic fibroblasts
NT: nontarget
WT: wild type

## 1 INTRODUCTION

Protein kinases are attractive candidates as targets for the development of new drugs due to the integral role they play in regulating key inflammatory signaling pathways that underpin a host of disease states.^1^ Members of the serine/threonine protein kinase family, such as c‐Jun N‐terminal kinase (JNK), have been under investigation in the cancer field due to the their essential roles in cell survival, growth, and death processes.^2^, ^3^ The major challenge these targets present in drug development is the high degree of structural similarity across family members that make specificity difficult to achieve without off targets and side effects.^4^, ^5^

The JNK family consists of three proteins, JNK1, JNK2, and JNK3, with JNK1 and JNK2 expressed ubiquitously while JNK3 expression is restricted to the brain, heart, and testis.^6^ JNK1 and JNK2 has recently been demonstrated to function independently therefore before success can be made in developing more selective drugs that target this pathway, there has to be a clearer understanding of the JNK1‐ and JNK2‐specific roles. While inhibitors exist that suppress the JNK pathway, so far they display poor potency^7^ and are nonselective.^8^ Many studies investigating JNK as a target have used pharmacological inhibition of the signaling pathway by SP600125. In addition to targeting JNK1, JNK2, and JNK3, SP600125 has been previously demonstrated to suppress the activity of a number of other serine/threonine kinases,^9^ which makes SP600125 an inadequate tool for JNK1‐ and JNK2‐specific roles to be determined. Success in this area has been best illustrated in studies using JNK1 and JNK2 knock out mouse models. Studies using mouse embryonic fibroblasts (MEFs) derived from JNK knockout mice have revealed differences in JNK isoform function. Sabapathy et al have demonstrated that JNK1 activated the transcription factor c‐Jun and played a role in promoting proliferation of MEFs, whereas JNK2 functioned as a negative regulator by promoting c‐Jun degradation and slowing down fibroblast proliferation.^10^ JNK1 and JNK2 have also been shown to function differently in cell death induced by ultraviolet (UV) radiation.^11^ JNK1 knockout resulted in partial protection against UV radiation in MEFs, whereas JNK2^−/−^ MEFs behaved similarly to wild type (WT). These findings demonstrate that JNK isoforms can function differently and independently in both cell death and cell growth processes in MEFs, however, a detailed understanding of JNK1 and JNK2 function needs to be more widely explored in human cell models. This has been best exemplified in the cancer field where different roles for JNK isoforms have been demonstrated using a variety of different tools.

In hepatocellular carcinoma (HCC), JNK has been linked to both the pathogenesis and poor prognosis of the disease.^12^ In this study, lentiviral approaches were used to stably knockdown JNK1, JNK2, and JNK1/2 in the HuH‐7 HCC cell line to investigate the effects of JNK knockdown on cell proliferation. JNK1 and JNK1/2 knockdown produced a decrease in proliferation, whereas JNK2 knockdown had no significant effect on proliferation which translated to the development of smaller tumors when implanted into nude mice, clearly linking JNK1 but not JNK2 to the tumorigenesis of HCC cells. Other studies have used the JNK inhibitor, SP600125, to pharmacologically inhibit JNK activity to implicate this pathway in breast cancer cell models.^13^, ^14^ Studies carried out in the MCF‐7 breast cancer cell line have shown that the JNK pathway is required for cell death in response to UV^15^ and taxol^16^ and also for MCF‐7 proliferation and cell cycle progression.^15^ Given the selectivity issues of SP600125, the roles attributed to JNK1 and JNK2 remain unclear, therefore JNK as a target in this context needs to be investigated further using more selective tools.

Here we explore JNK1 and JNK2 function in breast cancer cell lines using lentiviral shRNA to selectively knock down these targets. In order for comparisons to be drawn, SP600125 will be used to ensure that previously published data can be reproduced in the MCF‐7 cell line.

## 2 MATERIALS AND METHODS

### 2.1 Lentivirus production and lentiviral infection

shRNA targeting JNK1 (TAGATGCATCTATTACCAG), JNK2 (TCATGATCTAGCTCCATCT) and nontargeting control (ATCTCGCTTGGGCGAGAGTAAG) cloned into a pGIPZ lentiviral plasmid were purchased from Dharmacon™. These transfer vectors (2 μg) were cotransfected into HEK‐293T cells together with 2 μg of packaging vector pCMV—dR8.2 dvpr (Addgene), 2 μg of envelope vector pCMV‐VSV‐G (Addgene), and 12 μL of Turbofect (Thermoscientific) transfection reagent in media lacking antibiotics. After 12‐16 hours, media were replenished with complete media. After a further 24 hours, the media, now containing lentivirus particles, were harvested and the media were replenished with a final harvest carried out after a further 24 hours. Lentivirus was concentrated 100× using Lenti‐X‐concentrator (Takara Bio USA Inc.). Lentiviruses were referred to as NT for nontargeting and JNK1 and JNK2 for the targeted silencing of JNK1 and JNK2. For lentiviral infection, MCF‐7 cells were grown to 50% confluency and infected with either NT, JNK1, JNK2, or co transfected with JNK1 and JNK2 lentivirus for 48 hours. For selection, media were replaced with complete media containing 1 μg/mL of puromycin for a further 48 hours or until all uninfected cells died. Cells were selected every passage to maintain stable lines.

### 2.2 Cell culture

The breast cancer MCF‐7 cell lines were maintained in Dulbecco's modified Eagle's medium containing 10% (v:v) fetal calf serum, 27 mg/mL glutamine, and penicillin/streptomycin (250 units/mL and 25 mg/mL, respectively) at 37°C in a humidified air containing 5% CO_2_. For experiments, cells were seeded at specific densities: Western blot and FACS analysis (150 000 cells/mL), MTT (10 000 cells/mL), and clonogenics (120 cells/mL).

### 2.3 Stimulations for western blot analysis

Cells were starved for 24 hours and stimulated with 10% FCS for 1 hour or 30 J/m^2^ UV radiation for 24 hours before cells were harvested in 1× Laemmli Sample Buffer. For taxol experiments, cells were stimulated with 1% DMSO or 20 nmol·L^−1^ of taxol for 24 hours before cells were harvested in 1× Laemmli Sample Buffer. For SP600125 (Sigma‐Aldrich) experiments, cells were pretreated with 30 μmol·L^−1^ of SP600125 for 1 hour prior to stimulation with 10% FCS or 30 J/m^2^ UV radiation.

### 2.4 Western blot

For protein detection, cellular protein was subjected to 10% SDS‐PAGE and blotted onto nitrocellulose membrane. The membranes were blocked for nonspecific binding for 2 hours using 50 mmol·L^−1^ Tris buffer (pH 7.4), 150 mmol·L^−1^ NaCl, 0.1% (v:v) Tween‐20 (TBS‐T), containing 5% Bovine Serum Albium (BSA) (w:v). Blots were then incubated overnight in 0.5% BSA (w:v) TBS‐T with primary antibody. Blots were washed for 15 minutes in TBS‐T before being incubated for 2 hours in 0.5% BSA (w:v) TBS‐T with 1:7500 dilution of HRP‐conjugated secondary antibody. Blots were developed using ECL reagent followed by exposure to Kodak X‐ray film. Antibodies: pJNK 1:1500 (cell signaling technology #9251), pc‐Jun 1:1000 (Santa Cruz SC‐822), cleaved PARP 1:1000 (cell signaling technology #5625), JNK1 1:7500 (cell signaling technology #3708), JNK2 1:7500 (cell signaling technology #4672), and GAPDH 1:60000 (cell signaling technology #2118).

### 2.5 MTT

#### 2.5.1 Proliferation

On Day 1, cells from control, NT, JNK1, JNK2, and JNK1/2 cell lines were seeded onto 96‐well plates and incubated for 8 days at 37°C in a humidified air containing 5% CO_2_, media were replenished on day 4. On days 2, 4, 6, and 8, 10 μL of MTT (3‐(4,5‐dimethylthiazol‐2‐yl)‐2,5‐diphenyltetrazolium bromide) (Sigma–Aldrich) was added to each well and cells were incubated for 2 hours. Media were then removed from the wells and 100 μL of DMSO was added to dissolve the purple formazan product. Absorbance (570 nm) was then measured to estimate cell viability.

For experiments with SP600125, MCF‐7 cells were treated with a lower concentration of SP600125 (20 μmol·L^−1^) due to repeated treatment after the initial measurement on day 2 and on days 4 and 6. Controls of media alone and 1% DMSO were also replenished.

#### 2.5.2 Cell viability

Cells were seeded onto 96 well plates and stimulated for 24 hours with either 1% DMSO or 20 nmol·L^−1^ taxol. 10 μL of MTT (3‐(4,5‐dimethylthiazol‐2‐yl)‐2,5‐diphenyltetrazolium bromide) (Sigma‐Aldrich) was added to each well and cells were incubated for 2 hours. Media were then removed from the wells and 100 μL of DMSO was added to dissolve the purple formazan product. Absorbance (570 nm) was then measured to estimate cell viability.

### 2.6 Clonogenic assay

Cells were seeded onto 60 mm^2^ dishes and incubated overnight at 37°C in a humidified air containing 5% CO_2_. Cells were starved for 24 hours and then exposed to 10 J/m^2^ of UV radiation. Media were replaced with complete media and plates were incubated for 12‐15 days, with media replenished on days 5 and 10. After incubation, the media were removed; cells were washed with PBS, fixed with methanol, and stained with Giemsa for 20 minutes. After stain was removed, formed colonies were counted manually and survival fractions were calculated. For SP600125 experiments, cells were pretreated with 30 μmol·L^−1^ of SP600125 1 hour before exposure to UV radiation, the media remained on the cells 6 hours after exposure and were then replaced with complete media and followed the same course as previously described.

### 2.7 FACS

#### 2.7.1 Cell cycle

For cell cycle progression analysis, MCF‐7 cells were starved for 24 hours and then replenished with complete media for 24 and 48 hours with samples collected at each stage. Cells were trypsinized and fixed in 70% ethanol in PBS at 4°C for 20 minutes—2 days. Cells were then washed in PBS, centrifuged at 1800g for 10 minutes and then resuspended in 250 μL of PBS. 50 μg/mL of RNAse A (Sigma‐Aldrich) was added to each sample and incubated for 30 minutes at 37°C. 50 μg/mL of Propidium Iodide (Sigma‐Aldrich) was added to each sample and tubes were vortexed before analysis. Samples were read in FACSCanto flow cytometer and data were analyzed using FACS Diva software (FACS scan, Becton Dickinson, Oxford, UK). A total of 10000 events were measured per sample and gating was determined using PI‐stained populations. Cell cycle events were gated on G1, S, G2/M, and sub G1and the % of total events in each phase was measured. For SP600125 experiments, cells were treated with 30 μmol·L^−1^ of SP600125 or 1% DMSO when media were replenished after 24 hours of starvation. For taxol experiments, cells were collected the same as above after 24 or 48 hours treatment with 20 nmol·L^−1^ of taxol or 1% DMSO.

#### 2.7.2 Apoptosis

After treatment with 1% DMSO or 20 nmol·L^−1^ taxol for 48 hours or 500 μmol·L^−1^ H_2_O_2_ for 24 hours as positive control, cells were trypsinized and washed twice in PBS. As suggested by company guidelines, cells were resuspended in 100 μL of 1× Annexin V binding buffer (BD bioscience) and stained with 5 μL of APC‐Annexin V (BD bioscience) for 15 minutes at room temperature. Samples were read in FACSCanto flow cytometer and data were analyzed using FACS Diva software (FACS scan, Becton Dickinson, Oxford, UK). A total of 10 000 events were measured per sample and gating was determined using APC‐Annexin V individually stained cells and data were represented as the % of Annexin V‐positive cells.

### 2.8 Data analysis and processing

All statistics were calculated using GraphPad Prism version 5.01. Datasets were analyzed for statistical significance using either a one or two‐way analysis of variance (ANOVA). *P* values <.05 were considered significant and means +/− standard errors of the mean (SEM) are depicted in all figures.

## 3 RESULTS

### 3.1 Inhibition of JNK by SP600125 causes cell cycle arrest and a reduction in cell proliferation

**Figure 1:**
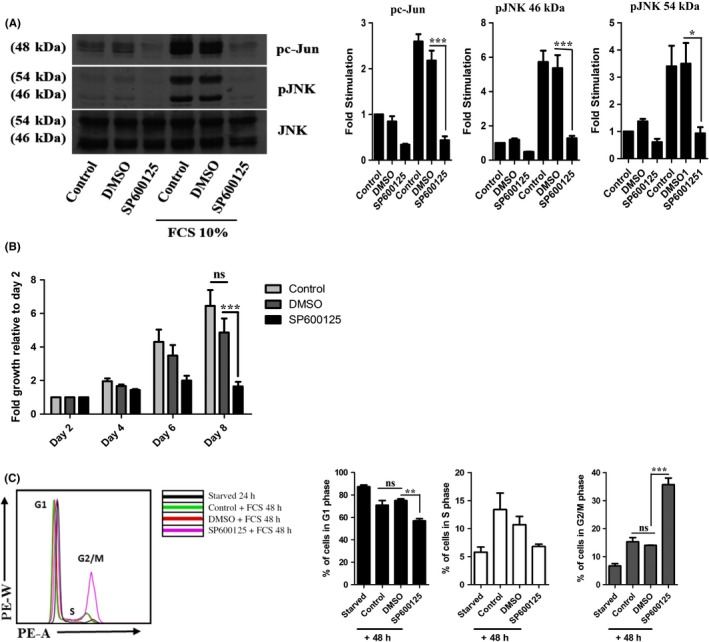
Inhibition of JNK by SP600125 causes cell cycle arrest and a reduction in cell proliferation. Cells were treated with media alone, 1% DMSO or SP600125 as stated in methods and the effects of JNK inhibition on (A) pc‐Jun expression, (B) proliferation, and (C) cell cycle progression were analyzed. Data represent the mean ± SEM of 3 independent experiments where *P < .05, **P < .01 and ***P < .001

Previous studies investigating JNK function in MCF‐7 cells have used transient methods of inhibition^15^ or knockdown^17^ to analyze their effects on cellular processes. The JNK inhibitor SP600125 is commonly used, therefore we initially sought to confirm if SP600125 could inhibit JNK signaling in MCF‐7 cells in our studies and investigate the effect of inhibition on cell growth. Treatment with 10% FCS increased the expression of pc‐Jun by 1.59‐fold ± 0.16, pJNK (54 kDa) by 2.39‐fold ± 0.75 and pJNK (46 kDa) by 4.72‐fold ± 0.65 when compared with nontreated cells (Figure 1A). Similar results were produced by cells that were pretreated with 1% DMSO (1.17 ± 0.21, 2.49 ± 0.76, and 4.36 ± 0.76, respectively), however, pretreatment with SP600125 reduced the levels of phosphorylated c‐Jun and JNK back to basal levels after stimulation with 10% FCS (Figure 1A). Proliferation was also reduced over 8 days when compared with the DMSO‐treated cells (Figure 1B). Control and DMSO‐treated cells grew steadily over the 8 days with an average fold growth of 3.4 ± 1.2 and 2.7 ± 0.9, respectively (Figure 1B). While treatment with SP600125 inhibited cell growth to 1.5‐fold ± 0.2, suggesting that JNK is involved in MCF‐7 cell growth. To understand how JNK inhibition may be preventing MCF‐7 growth, the effects of SP600125 on cell cycle progression was investigated using FACS analysis (Figure 1C). Treatment with SP600125 produced an arrest which was represented by an increase of 21.7% ± 2.3 in the population of cells in the G2/M phase of the cell cycle when compared with DMSO‐treated cells. These results coincide with those published by,^15^ thus confirming reproducibility of this JNK phenomenon in MCF‐7 cells.

### 3.2 Generation of MCF‐7 cell lines containing JNK individual isoform knockdown

**Figure 2:**
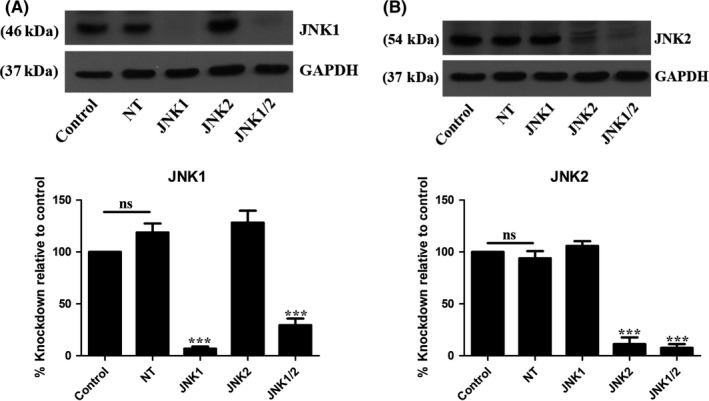
Confirmation of JNK1, JNK2, and JNK1/2 knockdown in MCF‐7 cell lines. MCF‐7 cells were collected at each passage and expression levels were analyzed using Western blot. Results show representative blot of (A) JNK1 and (B) JNK2 knockdown. Data represent the mean ± SEM of 4 independent experiments where ***P < .001

After confirming that inhibition of JNK by SP600125 influenced cell cycle progression and proliferation, we next investigated which JNK isoforms played a role in these processes. The Lentiviral delivery method of shRNA has been used to knockdown JNK isoforms in different cell lines including human epithelial,^18^ human liver cancer,^19^ and mouse mammary tumor cells.^20^ As this has allowed differences in isoform function to be determined in these studies, we used lentiviral shRNA to generate MCF‐7 cell lines containing stable knockdown of JNK1, JNK2, and JNK1/2. Western blotting experiments confirmed that protein levels were reduced by 93.21% ± 2.03 and 88.76% ± 6.49 for JNK1 and JNK2, respectively, in lines containing single knockdown and 70.54% ± 6.39 (JNK1) and 92.47 ± 3.65 (JNK2) in double knockdown lines when compared with control cells (Figure 2).

### 3.3 Knockdown of JNK isoforms has no effect on MCF‐7 proliferation, cell cycle progression or phosphorylation of c‐Jun

**Figure 3:**
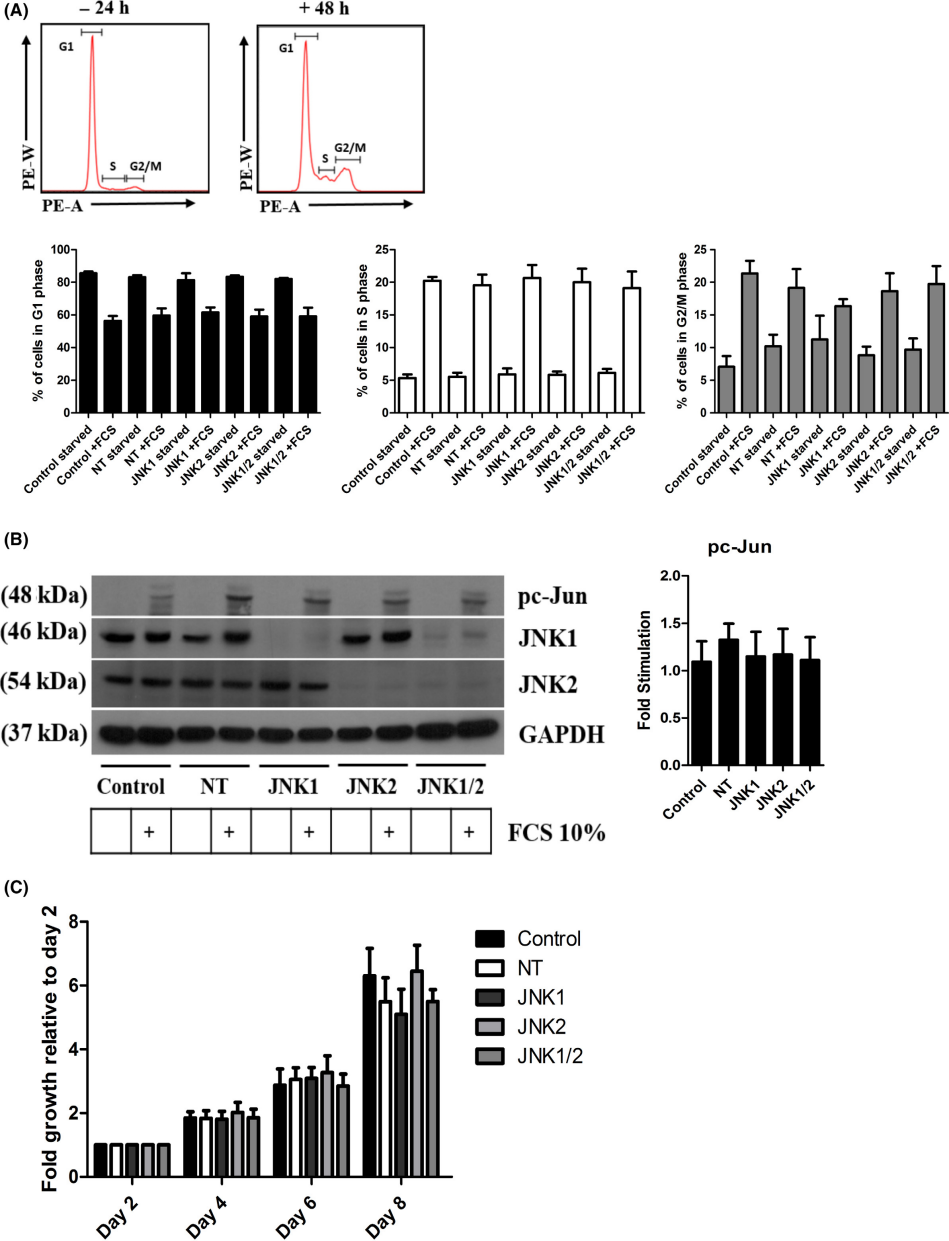
Cell cycle progression, proliferation, and activation of c‐Jun is independent of JNK. (A) Cells were starved for 24 hours and then replenished with complete media for 48 hours, samples were analyzed by FACS for the % of cells in G1, S, and G2/M of the cell cycle. (B) The number of viable cells measured by MTT represented as a fold increase relative to the number of viable cells on day 2. (C) Cells were starved for 24 hours, stimulated with 10% FCS for 1 hour and then collected and expression levels of pc‐Jun, JNK1, JNK2, and GAPDH were analyzed by Western Blot. Data represent the mean ± SEM of 3 independent experiments

After cell lines were successfully generated and knockdown was confirmed, these cells were used to investigate the effects of JNK isoform knockdown on cell cycle progression using FACS analysis. Our data show that over 48 hours there was a decrease in the percentage of cells in the G1 phase of the cell cycle (control 29.12% ± 3.0, NT 23.43 ± 4.3, JNK1 19.7 ± 3.1, JNK2 24.37 ± 4.2, and JNK1/2 22.83 ± 5.2) and an increase in the S (control 14.90% ± 0.6, NT 14.04 ± 1.6, JNK1 14.77 ± 2.0, JNK2 14.20 ± 2.1, and JNK1/2 13.0 ± 2.5) and G2/M phase (control 14.67% ± 1.9, NT 8.76 ± 2.8, JNK1 5.06 ± 1.1, JNK2 9.84 ± 2.7, and JNK1/2 10.03 ± 2.6) (Figure 3A). Interestingly there were not any major differences observed between the cell lines, including the control and nontarget (NT) lines (Figure 3A). There was also no cell cycle arrest produced by the knockdown of JNK demonstrating contrasting results when compared with the experiments using SP600125. Similarly, JNK isoform knockdown had little effect on the phosphorylation of cJun (Figure 3B) and the proliferation of MCF‐7 cells (Figure 3C) where all cells produced a fold growth of between five and sevenfold with slight variation between experiments taken into consideration. Interestingly, when experiments using SP600125 were repeated in JNK isoform knockdown cells, loss of JNK expression did not alter the effects produced by SP600125 (Figure S1).

### 3.4 UV‐induced cell death is independent of JNK in MCF‐7 cells

**Figure 4:**
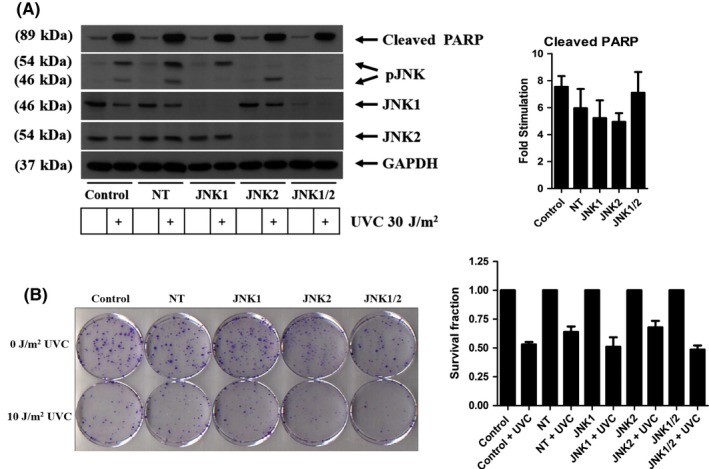
UV‐induced cell death is independent of JNK. (A) Cells were starved for 24 hours, exposed to 30 J/m^2^ of UV radiation for 24 hours and then samples were collected and expression levels of cleaved PARP, pJNK, JNK1, JNK2, and GAPDH were analyzed. (B) Cells were starved for 24 hours, exposed to 10 J/m^2^ UV radiation, cells were replenished with complete media and incubated for 12‐15 days. A clonogenic assay was used to measure successful colonies formed for both exposed and unexposed cells. Data represent mean ± SEM of 3 independent experiments

Since the knockdown of JNK isoforms did not affect processes involved in cell growth, we wanted to investigate whether the same occurred when analyzing cell death. JNK has been previously demonstrated to play a key role in UV‐induced cell death in MEFs where knockout of JNK prevented cell death for up to 28 hours after exposure to UV radiation.^11^ Following serum starvation, MCF‐7 cells were treated with 30 J/m^2^ of UV radiation for 24 hours and the expression of cleaved PARP was measured (Figure 4A). Similar to the cell growth experiments, knockdown of JNK isoforms did not produce a great change in cleaved PARP expression with all cell lines producing a fold stimulation of between five and eightfold (Control 7.54 ± 0.79, NT 5.95 ± 1.42, JNK1 knockdown 5.22 ± 1.31, JNK2 knockdown 4.93 ± 0.65, and JNK1/2 knockdown 7.1 ± 1.53. This was confirmed by clonogenic assay where cells were exposed to a lower dose of 10 J/m^2^, immediately replenished with complete media and then incubated for a longer period of 12‐15 days. Again, all cell lines produced a similar survival fraction (0.5‐0.6) after treatment with UV (Figure 4B), suggesting that JNK does not play a role in UV‐induced cell death in MCF‐7 cells.

### 3.5 Taxol‐induced cell cycle arrest and death is independent of JNK

**Figure 5:**
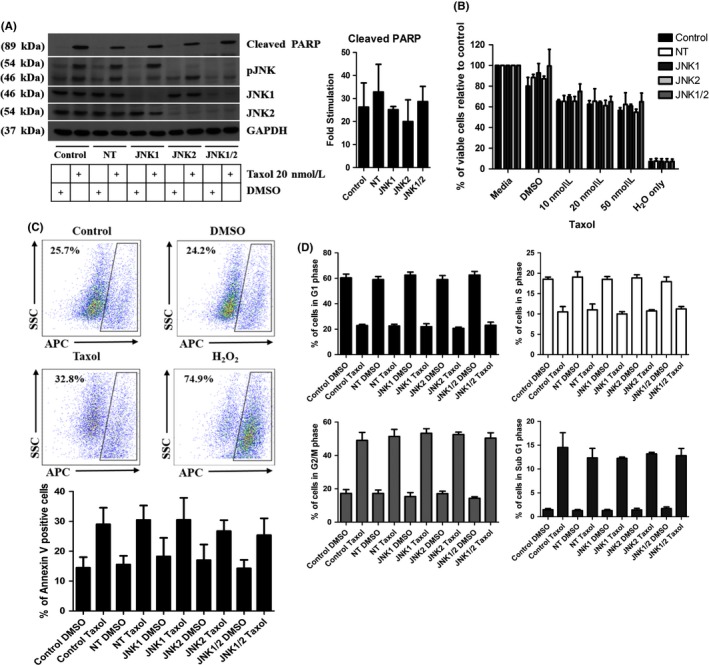
Taxol‐induced cell death is independent of JNK. Cells were treated with DMSO or taxol for (A, B, D) 24 hours or (C) 48 hours and samples were collected and analyzed for (A) expression levels of cleaved PARP, pJNK, JNK1, JNK2, and JNK1/2 by Western Blot. (B) Cell viability using an MTT assay, (C) Apoptosis using FACS analysis of Annexin V positive cells and (D) cell cycle arrest induced by taxol using FACS analysis gated on PI positive cells represented as the number of positive cells in G1, S, G2/M, and Sub G1 phase of the cell cycle. Data represent the mean ± SEM of 3 independent experiments

To understand if JNK played a role in cell death produced by other stimulants, we treated MCF‐7 cells with 20 nmol·L^−1^ of taxol for 24‐48 hours and assessed the effects of JNK isoform knockdown on cell death. As expected, taxol‐induced an increase in the expression of cleaved PARP by 26.2 fold ± 10.56 (control), 32.78 fold ± 12.05 (NT), 25.15 fold ± 1.38 (JNK1), 19.98 fold ± 9.43 (JNK2), and 28.62 fold ± 6.61 (JNK1/2) (Figure 5A) after 24 hours, although the fold stimulation varied between the different cell lines, these differences were insignificant when compared with control and NT samples. Taxol also produced a decrease of 37.59% ± 3.59 (control), 34.57% ± 12.10 (NT), 35.65% + 1.10 (JNK1), 38.74% ± 5.06 (JNK2), and 35.09% ± 5.27 (JNK1/2) in the number of viable cells after 24 hours (Figure 5B) and an increase of 14.56% ± 5.54 (control), 14.94% ± 4.84 (NT), 12.27% ± 7.39 (JNK1), 9.7% ± 3.69 (JNK2), and 11.17% ± 5.60 (JNK1/2) in the percentage of apoptotic cells after 48 hours of treatment with taxol (Figure 5C). Similar to the UV experiments, knockdown of JNK did not reverse cell death induced by Taxol over 24 or 48 hours. Finally, the role of JNK isoforms in taxol‐induced cell cycle arrest was investigated. As expected, treatment with taxol for 24 hours caused the percentage of cells in the G2/M phase of the cell cycle to increase by 31.73% ± 4.8, 34.1% ± 4.26, 37.87% ± 2.77, 35.40% ± 1.42, and 36.07% ± 3.20 for control, NT, JNK1, JNK2, and JNK1/2 knockdown lines, respectively. Again, this was the similar across all 5 cell lines confirming that JNK does not play a role in taxol‐induced cell death in MCF‐7 cells.

## 4 DISCUSSION

Extensive research has been carried out to demonstrate that the JNK pathway, and now more specifically the individual JNK isoforms themselves, play key roles in both cancer cell survival and cell death processes.^21^ The outcome of this signaling is determined by a number of variables including cell type,^22^ cell location,^23^ stimulant type,^24^ length of stimulation,^25^ and protein location.^26^ Due to the plethora of variables involved, characterization of the JNK pathway can be difficult as every model, cell type or technique can produce a different result. Many studies investigating JNK function to date have used pharmacological inhibition of the signaling pathway by SP600125. Although this inhibitor does block JNK signaling, it has also been reported to inhibit 13 other protein kinases, including AMPK, CDK2, and SGK.^9^ Although JNK is an attractive target, there is yet to be a successful drug developed which can inhibit this protein without off‐target effects.^8^ In order for progress to be made in this area, more selective approaches need to be adopted to ensure that the effects observed are due to JNK and not one of the many off‐targets inhibited by SP600125.

In our own investigation, we managed to recapitulate previously published findings using the SP600125 inhibitor. If the pharmacological data generated using SP600125 were to be solely relied upon, our data would similarly support a role for JNK signaling in MCF‐7 cells proliferation and cell cycle progression. However the data presented in our study highlight the critical need of adopting a more selective approach when interrogating the JNK pathway and not relying solely on the use of SP600125.

In this study, stable and selective lentiviral knockdown of JNK1 and JNK2 was achieved in MCF‐7 cells. Although consistent JNK isoform knockdown was achieved, no effect on cell death induced by UV radiation or taxol was observed. This was confirmed using a variety of approaches including cell viability assays, Western blotting, and FACS analysis. These results were surprising given that previous studies using SP600125 had proposed that JNK play a role in both UV^15^, ^27^ and taxol‐28 induced cell death in MCF‐7 cells. Similarly, knockdown of JNK1 or JNK2 in our studies had no effect on cell growth. This was observed for both proliferation and cell cycle progression experiments. Again, these results were surprising given that JNK signaling has been linked to both proliferation^17^ and cell cycle progression^15^ in MCF‐7 cells when treated with SP600125. JNK is a known regulator of c‐Jun in a number of different cell lines.^10^, ^29^ While loss of JNK in our studies resulted in inhibition of JNK phosphorylation, this did not impact phosphorylation of c‐Jun. Interestingly, inhibition of JNK with SP600125 clearly inhibited MCF‐7 proliferation and phosphorylation of c‐Jun, producing contrasting results to experiments conducted using lentiviral knockdown of JNK.

**Figure 6:**
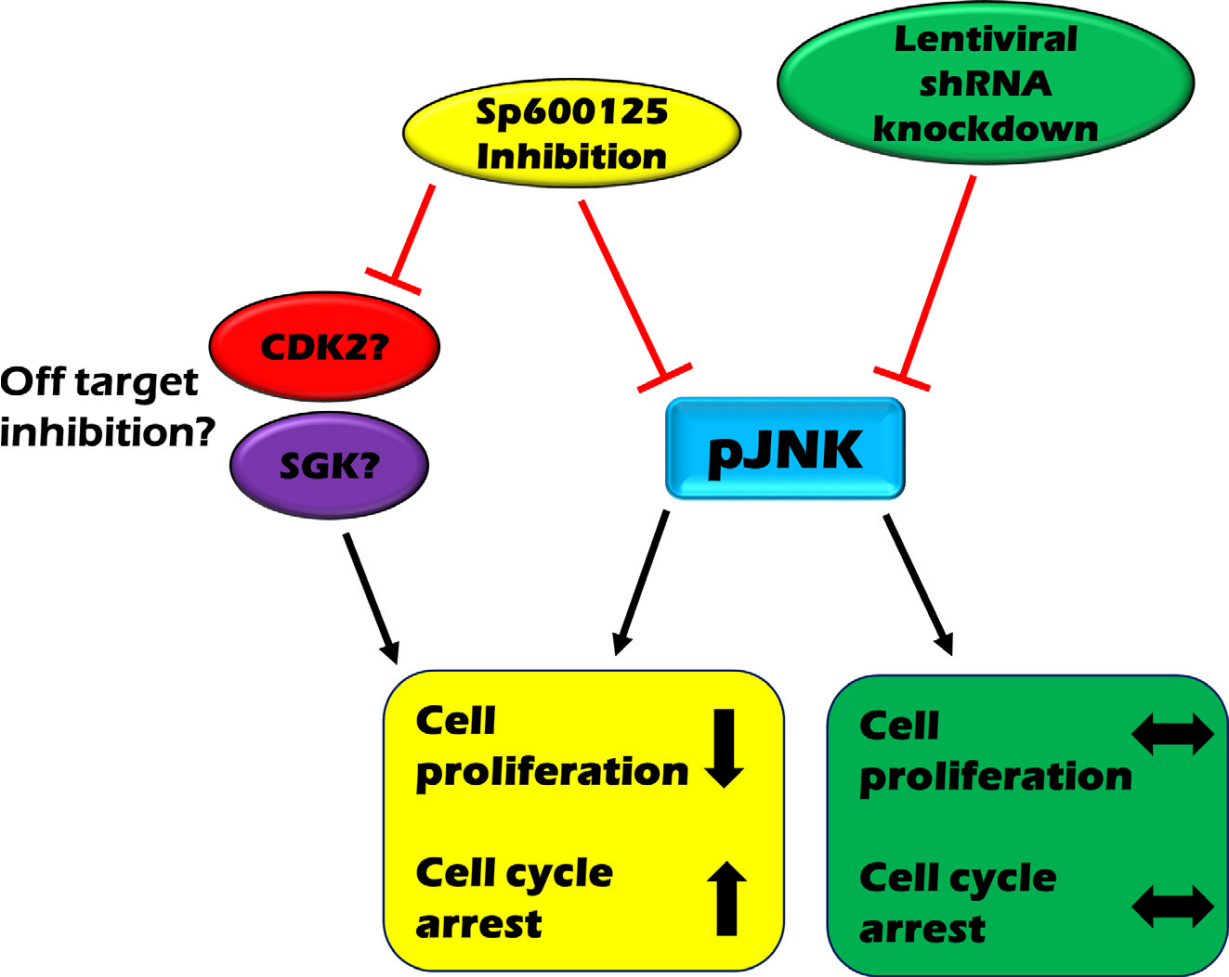
Schematic of SP600125 inhibition vs shRNA knockdown of JNK in MCF‐7 cells. Treatment of MCF‐7 cells with SP600125 produced inhibition of JNK phosphorylation, an increase in cell cycle arrest and inhibition of cell proliferation. In contrast shRNA knockdown of JNK inhibited the phosphorylation of JNK however this had no effect on cell cycle progression or proliferation of MCF‐7 cells. SP600125 has been demonstrated to inhibited kinases CDK2 and SGK 9 both of which have been shown to play a role in breast cancer cell proliferation and cell cycle progression^29^, ^30^ and therefore this could be an alternative path as to which SP600125 can elicit the effects produced in this study

The fact that both methods of protein targeting resulted in inhibition of JNK phosphorylation suggests that the cellular events downstream of UV and taxol are independent of JNK. In light of the numerous off‐targets cited for SP600125, we cannot rule out the possibility that inhibition of these kinases may be a contributing factor to the contrasting results produced in this study (summarized in Figure 6). When combination experiments were carried out using SP600125 in JNK isoform‐specific knockdown cell lines, SP600125 clearly inhibited MCF‐7 proliferation and cell cycle progression. This suggests that the actions of SP600125 may be independent of JNK. Two such proteins inhibited by SP600125, namely SDK^30^ and CDK2,^31^ have been linked to breast cancer cell proliferation and cell cycle progression. Inhibition of these kinases may be contributing to the pronounced effects observed in SP600125 studies.

Trying to determine JNK isoform function in cancer is very challenging due to the differences in JNK function between cancer types,^19^, ^32^ however, with more selective tools becoming available it should be possible. Studies using JNK knockout animals clearly present a good rationale for why targeting JNK isoforms may be of therapeutic value,^10^ however firm translation of these findings into human cell models is crucial. For example, JNK1 but not JNK2 was shown to be required for UV‐mediated death in MEFs.^33^ However, in this study JNK knockdown had no effect on MCF‐7 cell death induced by UV exposure. Differences in the translation of these events from mouse cells to human cell models may account for these conflicting findings. The use of JNK knockdown, or gene deletion approaches such as CRISPR, in other cell systems may help to shed some light on the role for JNK in human cell growth and death.

What is clear from our study is that any interpretations derived from studies that rely solely on the use of SP600125 need to be treated with caution when attributing the JNK pathway to MCF‐7 cell growth and death. Due to the sheer number of other serine/threonine kinases inhibited by SP600125, it would be wise to employ more selective ways to validate the role of JNK in cancer cell function. While shRNA approaches have their own set of challenges to ensure complete knock down of protein levels are achieved, the availability of gene editing approaches such as CRISPR will pave the way for a better fundamental understanding of the role of JNK (or any other target) in human cancer cell function. This will be essential if progress in the field is to be made and new targets are to be identified for anticancer therapies.

## DISCLOSURES

The authors have no conflict of interest to declare.

## AUTHOR CONTRIBUTIONS

Rachel Wood: conceived the project, carried out the majority of the experimental work and wrote the manuscript. Robin Plevin: conceived the project, provided primary supervision and read over the manuscript. Mark Barbour: assisted with experimental work and proofread the manuscript. Gwyn Gould: provided insight and expertise during the study. Margaret Cunningham: provided valuable feedback and direction on the experimental work and contributed to the writing of the manuscript.

## Supporting information


**Figure S1.** shRNA knockdown of JNK did not affect inhibition of proliferation and cell cycle progression by SP600125. Control, NT, JNK1, JNK2, and JNK1/2 knock down MCF‐7 cells were treated with media alone, 1% DMSO or SP600125 as stated in methods and the effects of JNK inhibition on (A) proliferation at 2, 4, 6, and 8 days and (B) cell cycle progression were analyzed. Data represent the mean ± SEM of 3 independent experiments
