# Supplementary figures and images for: Conflicting evidence for the role of JNK as a target in breast cancer cell proliferation: Comparisons between pharmacological inhibition and selective shRNA knockdown approaches
Source: Pharmacol Res Perspect. 2017 Dec 20;6(1):e00376. doi: 10.1002/prp2.376 (PMC5817830; doi:10.1002/prp2.376)

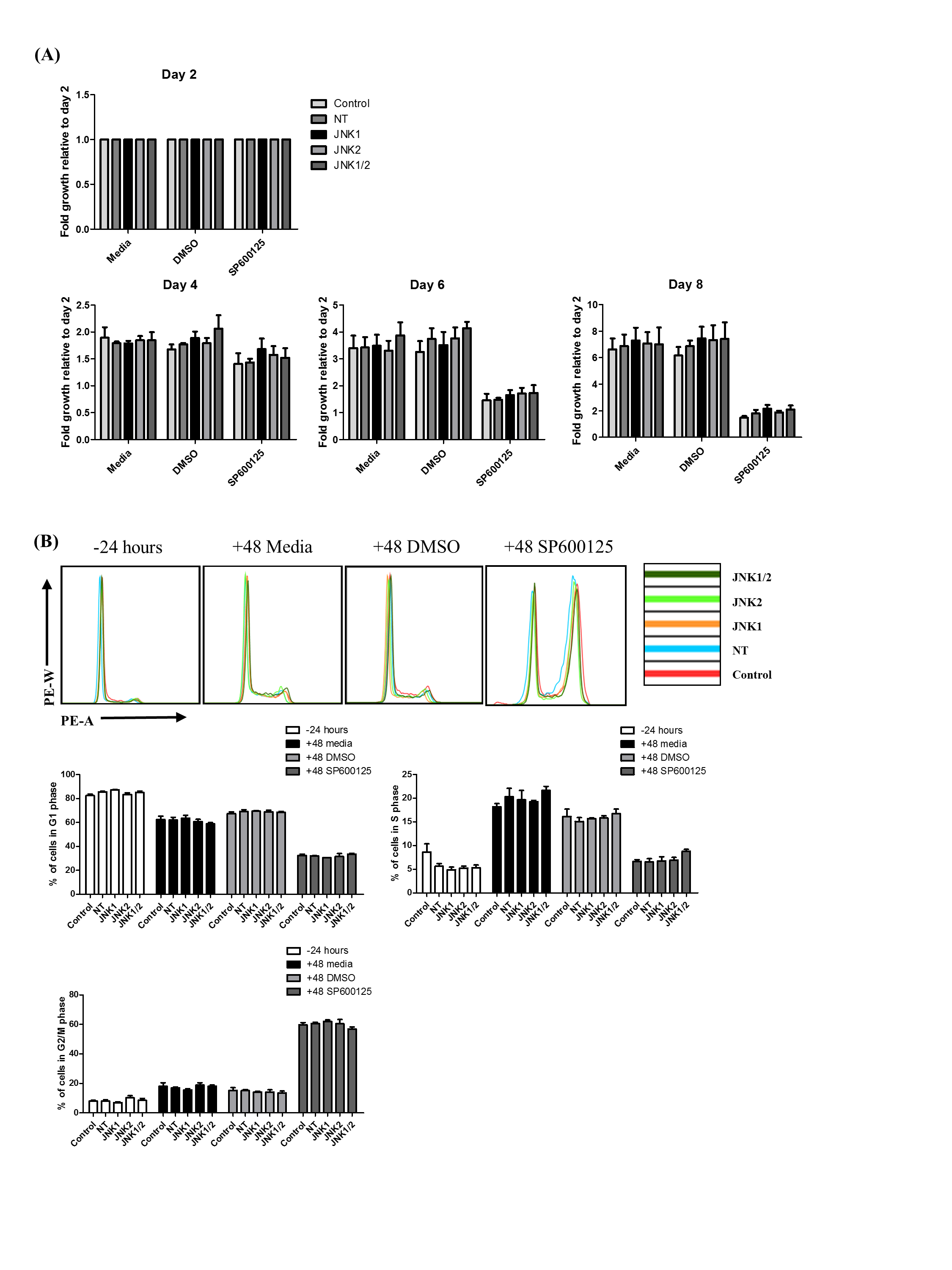

Supplement: Supplementary file 1 — Figure S1. shRNA knockdown of JNK did not affect inhibition of proliferation and cell cycle progression by SP600125. Control, NT, JNK1, JNK2, and JNK1/2 knock down MCF‐7 cells were treated with media alone, 1% DMSO or SP600125 as stated in methods and the effects of JNK inhibition on (A) proliferation at 2, 4, 6, and 8 days and (B) cell cycle progression were analyzed. Data represent the mean ± SEM of 3 independent experiments [file PRP2-6-e00376-s001.tif]
